# Dr. B. A. Morel on Mental Disorders
**Traité des Maladies Mentales*. Par le Dr. B. A. Morel, Médecin-en-Chef de l'Asile des Aléinés de Sainte-Yon (Seine Inférieure). Paris. 1860.


**Published:** 1860-04-01

**Authors:** 


### Art. VIII.—DR. B. A. MOREL ON MENTAL DISORDERS.*

Dr. B. A. Morel, who is perhaps best known to our readers by his *Traité des Dégénerescences Physiques, Intellectuelles et Morales de l'espèce humaine*, has very recently given to the world a treatise on mental maladies. Any work emanating from so highly competent a source would command attention, but this work merits particular notice for two reasons ; first, because the author has specially destined it for the use of physicians not alienists ; and second, because it is based upon a new classification of mental disorders.

The present work may be fittingly termed a complement to the treatise on degenerations already referred to. In that work, Dr. Morel endeavoured to show that, the psychical and moral as well as the physical deterioration observed in degenerated conditions of the human race, had fixed and definite characters in immediate relation with the causes which gave rise to these conditions. In the work before us he seeks to establish an intimate and constant relation between the form of mental alienation and the cause in which it originates, a natural extension of the idea just mentioned, and he proposes a classification based upon the etiology of mental disorders.

* *Traité des Maladies Mentales.* Par le Dr. B. A. Morel, Médecin-en-Chef de l'Asile des Aléinés de Sainte-Yon (Seine Inférieure). Paris. 1860.

We purpose to lay before our readers some account of the nature and mode of formation of this classification, and an estimate of the effect it will probably exercise on the study of mental disorders, if it be accepted by the profession.

In the first place it is needful clearly to distinguish between the *necessary* and *conditional* causes of insanity. Thus, for example, meringitis, phthisis, pneumonia, typhoid fever, hypertrophy of the heart, &c., moral impressions, such as fear, love, exaltation of religious sentiment, all the passions, in short, can occasion insanity, but not necessarily so. All who are exposed to these causes do not become insane, or not even threatened with insanity. It is only on *condition* of a *predisposition* which determines the action of a particular cause in a special direction, that any of the so-called *determining* or *occasional* causes give rise to insanity. The causes called specific, for example, alcohol, opium, &c., and certain idiopathic cerebral affections, alone have the power of producing *necessarily* permanent disturbance of the intellectual faculties under certain given circumstances.

Now it is requisite that divers elements, which participate equally of the physical and the moral nature of man, be called into action under the influence of the predisposition, and should concur with the occasional cause in order that a *new morbid type* or *a particular form of alienation* be formed, which shall stamp all the insane which are attached to this form with a *common character*.

When, therefore, Dr. Morel lays down the law that an *intimate* and *necessary relation exists between the form of alienation and the nature of the cause which gives rise to it,* he is not to be understood as implying that this relation is established under the influence of the least cause, which in inducing the elements of delirium, may ultimately lead to alienation. He holds that the cause cannot be separated either from the individual predisposition, or from the *functional disorder,* or from the *lesion* that the cause determines in the organism. Three elements are indeed necessary for the realization of a particular form of insanity, to wit, the *predisposition,* the *occasional cause,* and the *functional disorder* or *lesion.*

The further development of Dr. Morel's theory of causation will scarcely bear abstraction, therefore we give it in full :—

"If the predisposition does not exist, the occasional cause may certainly induce a disturbance of the intellectual functions, a general or systematic delirium, durable or transitory, an alienation in short, but rarely will this delirium have the character which is observed in particular or essential forms of insanity. Moreover, the prognosis will be all the more favourable because no predisposition exists, and because the state of the patient presents generally conditions of acuteness.

"If the cause communicates to the organism but a passing impression, the delirium which may be occasioned by it will be transitory and fugacious. It is only on condition of originating a durable disorder in the organism, of determining a special lesion of the nervous centres, that the delirium, at first transitory and ephemeral, advances presently to proportions which give it an entirely different character, and which constitute a special form of insanity.

"It is then permanent or durable, general or systematic. It is developed according to laws fixed and invariable; it derives the elements of its activity, of its mode of production, both in the nature of the cause, and in the spontaneity of the intelligence, and in the gravity and progress of the functional disorder or of the organic lesion; it begets insensate determinations, acts of a fatal and irresistible character. It is in short the index of insanity, so called, of that state which, in a psychological point of view, is not only the expression of the general sufferings of the organism, but of this or that suffering in relation with the nature of the cause and that of the lesion. It is in this point of view only that I am able to say that intimate and necessary relations are established between the nature of the cause and this or that form of alienation.

"If it were otherwise, and the forms of insanity were developed in an invariable manner under the influence of the least determining or occasional cause, these forms would be innumerable, and every attempt at classification would become impossible in presence of the multitude of causes which would have the fatal privilege of creating forms of alienation in relation with the specific nature of their action.

"It remains then but to designate the form of insanity by the name of the determining or occasional cause, and we shall immediately see how many errors of diagnosis and prognosis would be occasioned. Does not experience prove to us daily that we are almost invariably induced to attribute to the *determining cause* the part of *final cause*, and to neglect thus the real point of departure of the evil; in other words, the real point of departure of that cause which gives to insanity its *particular form*, which does not permit us to confound one variety of alienation with another, and the insane appertaining to one class with the insane belonging to another?

"It is then indispensable that the cause which conducts to insanity, to that exceptional state which makes man different from himself, it is indispensable, I say, that this cause should derive the elements of its activity in an order of facts or of phenomena which are engendered and which dominate in such a fashion, that if nothing is opposed to this generation and to this reciprocal dependency, there will result from it determined, fixed, and invariable effects, which must necessarily produce not only insanity with all its consequences, but *such a variety of insanity rather than such another*."—(pp. 250-2.)

The facts upon which this theory is founded are duly recapitulated by Dr. Morel, and upon the principles contained in it he has framed a classification of mental maladies, in which these affections are no longer characterized by the greater or less degree of exaltation which accompanies the delirium, as is observed in the states usually named *mania, melancholia, monomania, stupidity*. These phenomena are but symptoms which may be found in every variety of insanity :—

"My object," writes Dr. Morel, "is to seek in each form the characters which distinguish it from any other form, in such a manner, that the fundamental aspects once being given, we shall be able to recognise to what nosological variety in alienation belongs the individual who betrays either delirium of ideas or acts, or diverse lesions or disorders of the nervous functions."—(p. 258.)

Upon the etiological basis set forth, Dr. Morel divides mental disorders into six principal groups, each of which has several subdivisions. The following is a summary of this classification .—

1st Group.—*Hereditary alienations.*—This group is subdivided into four classes.

(*a*) The *first class* includes those in whom the nervous temperament is a congenital fact, in virtue of hereditary transmissions excessively varied. These individuals are more apt than others to be attacked with insanity.

(*b*) The *second class* contains those in whom hereditary transmission is revealed by psychical and physical phenomena, which approximate the insane of this category to a type that may be recognised by the following characters :—

"In the course of their existence, insanity is manifested among them much more by delirium of acts than words. They are distinguished by their eccentricities, by incoherence, singularity, and often even by the profound immorality of their actions. Certain remarkable intellectual qualities do not exonerate them from the impossibility of directing simultaneously their faculties towards a wise and useful end. Their creations are rare and commonly their inventions do not fructify. They are partial geniuses, and notwithstanding certain brilliant manifestations, they are struck with intellectual and sometimes even physical sterility.

"In this class are placed a multitude of individuals who indulge in chimerical projects, reformers of the human species, Utopianists of every kind, inventors whose discoveries are impossibilities, or who pursue the verification of insoluble problems (*monomanias* of certain authors).

"The dangerous acts which they commit in the paroxysms of their insanity, their *instinctively* bad tendencies, necessitate often the intervention of authority, and their sequestration. Their accessions of mania are of short duration, and in the remissions they manifest to observation the essential characters of their disease : systematic delirium, with haughty tendencies, without general paralysis. They astonish those who notice them but superficially by the apparent lucidity of their reason (*mania raissonnante* of authors, *moral insanity* of the English.")—(p. 259.)

(*c*) The *third class* forms a transition series between the individuals of the second and fourth classes.

In this, the third class, the signs of hereditary taint show themselves at a very early period, even at the most tender ages, by intellectual inertia, and excessive depravity of the moral disposition :—

"Their innate tendencies to evil have caused them to be characterized as *instinctive maniacs*. Incendiarism, robbery, vagabondage, precocious propensities for debauchery of all sorts, form the sad balance-sheet of their moral existence, and these unfortunates, who most commonly *have not been fecundated either in respect to physical or moral well being in humanity*, and who are in consequence the most direct representatives of hereditary transmissions of an evil nature, people in the greatest proportion our prisons and penitentiary institutions from their earliest childhood."—(p. 260.)

(*d*) The *fourth class* includes innocents, *imbeciles, idiots.*

2nd Group.—*Mental alienations from intoxication.*—The intimate relation which exists between the form of insanity and the nature of the cause is, perhaps, more clearly seen in the alienations produced by intoxicating substances than in any other varieties. As in the instance of the inordinate use of alcoholic liquors, certain toxic agents exercise a determinate effect upon the system, each giving rise to peculiar psychical disturbances. Whether man seeks to procure factitious sensations with alcohol, opium, or other inebriating substances; whether he be the victim of working in lead, mercury, phosphorus, or other metals ; or he suffer from the use of diseased nutriment, as spurred rye ; or from the air he respires being polluted, as in marshy districts ; or from the geological character of the soil, as in cretinism, we may look upon him as exposed to the influence of an intoxicating cause, and anticipate that the lesions of the nervous system will be in relation with the nature of the cause.

It may be said that these agents may cause rapid death preceded by more or less furious delirium, escaping from all classification in reference to insanity. This is true, but it is only in the chronic state that we shall have to study the different deliriums produced by these substances. The term *chronic alcoholism,* employed now to specify poisoning by alcohol, indicates in what manner this question ought to be posited.

Dr. Morel divides into three classes the morbid varieties included in the group of alienations caused by intoxication.

(*a*) The *first class* contains the varieties produced by *narcotic substances employed to procure factitious sensations, and by the deadly influence of certain forms of industry.* This class includes the effects of alcohol, opium, and other narcotics; also the morbid effects of working in lead, mercury, phosphorus, &c.

(*b*) The *second class* includes the effects of *an insufficient or deteriorated nutriment,* as, for example, the nervous epidemic *ergotism.*

(*c*) The *third class* includes the consequences of *paludal influences* and of the *geological constitution of the soil.*

3rd Group.—*Alienations determined by the transformations of certain neuroses.*

The alienation which is immediately developed from certain nevropathies, always reflects the fundamental character of the neurosis which has given rise to it. Dr. Morel divides the alienations of this group into three classes.

(*a*) *First class.* — *Hysterical alienation.*—In this form of alienation the phenomena characteristic of the hysterical temperament and state disappear, and are succeeded by certain symptoms every way special and peculiar. The greatest exaltation will succeed the most profound prostration. Hallucinations and bizarre sensations, extravagant delirium, rapid transitions from one nervous state to another, extraordinary remissions with apparent return to reason, and in some instances penchants to suicide, incendiarism, and to all kinds of acts of an evil nature; lastly, deplorable terminations in which human nature exhibits itself under the most degrading aspect,—these form the chief characters of hysterical insanity. Ecstasy, catalepsy, anæsthesia, in short, the phenomena that usually accompany hysteria are rarely observed. Hysterical insanity is, indeed, a transformed neurosis in the strictest sense of the term.

(*b*) *Second class.—Epileptic alienation.*

(*c*) *Third class.—Hypochondriacal alienation.*

This class is subdivided into three varieties.

(1) *Simple hypochondriasis.*

(2) *Hypochondriasis* characterized by *délire des persecutions.*

(3) *Hypochondriasis* distinguished by a general sentiment of well-being and delusions of grandeur, which supervene upon the habitual depression, exaggerated fears, and ideas of persecution observed in the second variety.

4th Group.—*Idiopathic alienations,*—the brain being directly compromised by affections which are peculiar to it, as periodic congestions, hæmorrhages, meningitis, cerebral softening, cerebral atrophy, traumatic lesions, blows, falls, &c. This group is divided into two chief classes.

(*a*) *First class,* distinguished by a *progressive enfeeblement or abolition of the intellectual faculties, a sequel of chronic maladies of the brain or its envelopes.*

(*b*) *Second class,* constituted by *general paralysis, or paralytic insanity with predominance of systematic, expansive delirium.*

"The determining causes of this affection are, as M. Parchappe has justly observed, of the number of those which provoke a strong and prolonged hyper-excitation of the brain: *sensual excesses*, and *especially the abuse of alcoholic drinks*, *of good cheer*, *of venereal pleasures*, *and intellectual excesses*, *represented particularly by prolonged watchings and pre-occupations with business*, *enterprises*, *works*, &c. . . But in order to form a just idea of this malady, so characteristic on account of the nature of the *ambitious delirium*, it is most necessary, after having studied the intimate relations which exist between the form of the insanity and the nature of the cause, to interpose the element of the *cerebral lesion.* In paralytic insanity, the lesion, which is nothing less than inflammatory softening of the cortical couch of the two cerebral hemispheres, stamps the malady with a character which is peculiar to it, and which certainly distinguishes it as one of the most marked morbid species met with in the nosological arrangement of mental maladies."—(p. 268.)

5th Group.—*Sympathetic alienations*, in which the morbid state of the brain is determined sympathetically by mischief existing in other parts of the frame—the brain being affected by *consensus*, as the older authors were accustomed to say. In this group Dr. Morel includes *erotomania* and *nymphomania*.

6th Group.—*Dementia.*—Dr. Morel believes that it will be useful to preserve this designation, because it has been adopted by the legislature, although in a sense differing from that accepted in medicine. Dementia is not, properly speaking, a primitive form, it is rather a terminal one. But as it happens that the numerous lunatics who have become demented, from whatever cause this may have arisen, form a numerous family, *of which all the members have common characters, and are recognised by certain internal and external signs*, Dr. Morel considers that the order and method which he seeks to introduce in mental maladies will not suffer from a classification which makes dementia one of the important varieties of insanity.

In concluding the explanation of the motives which have induced him to adopt the foregoing classification, Dr. Morel adds:—

"I shall be reproached without doubt for having blotted out two essential forms generally adopted, *mania* and *melancholia*. But I have already observed that mania (*exaltation*) and melancholia (*depression*) are symptoms which are met with in all the varieties of insanity, and which, consequently, do not constitute essential forms. In other respects I do not question the value of these designations which, indeed, ought to be preserved. The words *maniacal excitement*, *melancholic depression*, *mania* or *melancholy*, return frequently under my pen when it is necessary for me to describe the different phases of this or that variety of insanity which enters into the classification that I have adopted; but, I again add, these symptoms are only transitory phenomena which most commonly alternate with one another. Moreover, whenever I use the words *mania* and *melancholia*, it should be well understood that I refer but to certain phases of mental maladies or predominant symptoms of exaltation and depression, not intending to indicate particular forms of insanity. I describe but one of the symptoms of a determined form of mental alienation.

"I shall not enter into other considerations in order to justify the classification which I have adopted. Here, as in all the sciences of observation, the results must suffice to justify the method. If then the classification of mental maladies, *in their relations with the nature of the cause*, leads us to comprehend better the progress of these insidious maladies, and to give to our prognosis and treatment a more solid basis, I shall have attained the difficult aim which I have assayed.

"It is necessary to remember, however, that in my opinion, the action of the cause, in the generation of special forms of mental maladies, cannot be separated either from the individual predisposition, or the functional disorder which the cause creates, nor lastly, from the organic lesion, which sometimes is a primitive, sometimes a consecutive phenomenon."—(pp. 271, 272.)

In terminating this notice of Dr. Morel's classification, the most important and novel portion of his work, it is proper to remark that our analysis will hardly suffice to convey a correct notion of its true nature and significance, unless the reader have a previous acquaintance with our author's researches on *Degenerations*, the results of which, it may be added, are fully set forth in the present work.

And now, if we gauge the value of this classification by the measure which the author himself suggests, and which is supplied in his Treatise, we think that we are justified in the following conclusions:—

(1) That the classification is a far more healthy and useful one than that in ordinary use. It indoctrinates us from the first into the whole of those slighter forms of mental perturbation, which constitute the very substratum, so to speak, of fully-formed insanity, but which have hitherto been treated only incidentally, or in a disconnected manner, in treatises on mental disorders. It links straitly and systematically the special study of insanity proper to those great social questions of psychical and physical deterioration which meet us at every turn of life, and makes the study of the one the necessary complement of the other, or rather the study of the latter the necessary foundation for a right knowledge of the former. It substitutes for a classification based upon symptoms, and which chiefly consists of arbitrary *typical* forms, and is consequently artificial and provisional, one based upon the intimate relation between symptoms and causes, and hence natural.

(2) That etiology being made the essential principle of the classification, this gives us greater precision as well in our prognosis as our treatment of mental disorders, and more especially it enables us rightly to apprehend those vast questions involved in the general hygiene of mental disorders, and does not permit us, as we are too apt to do, to overlook these in the purely medical treatment of the disease.

It is probable that Dr. Morel's classification may be somewhat modified in form, or that it may be added to in process of time, but if adopted, the benefits arising from it, to which we have referred, would be direct and immediate.

We shall confine ourselves to this portion of Dr. Morel's work. If his name be not sufficiently well known among our readers, and form of itself a satisfactory guarantee for the manner in which the details of the book are worked out, we may assure them that these are in every respect admirable. The treatise is one, indeed, which, as a text-book for the student and the general practitioner, is unrivalled.

---

